# MAIT cell activation and dynamics associated with COVID-19 disease severity

**DOI:** 10.1126/sciimmunol.abe1670

**Published:** 2020-09-25

**Authors:** Tiphaine Parrot, Jean-Baptiste Gorin, Andrea Ponzetta, Kimia T. Maleki, Tobias Kammann, Johanna Emgård, André Perez-Potti, Takuya Sekine, Olga Rivera-Ballesteros, The Karolinska COVID-19 Study Group, Sara Gredmark-Russ, Olav Rooyackers, Elin Folkesson, Lars I. Eriksson, Anna Norrby-Teglund, Hans-Gustaf Ljunggren, Niklas K. Björkström, Soo Aleman, Marcus Buggert, Jonas Klingström, Kristoffer Strålin, Johan K. Sandberg

**Affiliations:** 1 Center for Infectious Medicine, Department of Medicine Huddinge, Karolinska Institutet, Karolinska University Hospital, Stockholm, Sweden.; 2 Department of Infectious Diseases, Karolinska University Hospital, Stockholm, Sweden.; 3 Department of Clinical Science, Interventions and Technology, Karolinska Institutet, Stockholm, Sweden.; 4 Perioperative Medicine and Intensive Care, Karolinska University Hospital, Stockholm, Sweden.; 5 Division of Infectious Diseases, Department of Medicine Solna, Karolinska Institutet, Stockholm, Sweden.; 6 Department of Physiology and Pharmacology, Karolinska Institutet, Stockholm, Sweden.; 7 Division of Infectious Diseases and Dermatology, Department of Medicine Huddinge, Karolinska Institutet, Stockholm, Sweden.

## Abstract

Severe coronavirus disease 2019 (COVID-19) is characterized by excessive inflammation of the lower airways. The balance of protective versus pathological immune responses in COVID-19 is incompletely understood. Mucosa-associated invariant T (MAIT) cells are antimicrobial T cells that recognize bacterial metabolites and can also function as innate-like sensors and mediators of antiviral responses. Here, we investigated the MAIT cell compartment in COVID-19 patients with moderate and severe disease, as well as in convalescence. We show profound and preferential decline in MAIT cells in the circulation of patients with active disease paired with strong activation. Furthermore, transcriptomic analyses indicated substantial MAIT cell enrichment and proinflammatory IL-17A bias in the airways. Unsupervised analysis identified MAIT cell CD69^high^ and CXCR3^low^ immunotypes associated with poor clinical outcome. MAIT cell levels normalized in the convalescent phase, consistent with dynamic recruitment to the tissues and later release back into the circulation when disease is resolved. These findings indicate that MAIT cells are engaged in the immune response against SARS-CoV-2 and suggest their possible involvement in COVID-19 immunopathogenesis.

## Innate-like rapid responders

Viral infections elicit host responses from conventional T cells, innate lymphoid cells, and innate-like lymphocyte subsets. Parrot *et al.* used blood from acute and convalescent COVID-19 patients to investigate how SARS-CoV-2 infection affects the innate-like mucosa-associated invariant T (MAIT) cells. Acute viral infection induced a profound decline in the number of blood MAIT cells and activation of the residual blood MAIT cells. The loss of circulating MAIT cells in acute COVID-19 patients coincided with enrichment of MAIT cells among T cells recovered from the respiratory tract. With convalescence, the number of blood MAIT cells and their activation status reverted toward normal. These findings indicate that circulating MAIT cells are mobilized early after SARS-CoV-2 infection and may contribute to both resolution and exacerbation of COVID-19–associated pneumonia.

## Teaser

MAIT cell activation and decline in blood are associated with COVID-19 severity, features that dynamically recover in convalescence.

## INTRODUCTION

Severe acute respiratory syndrome (SARS) coronavirus-2 (SARS-CoV-2) causes viral pneumonia and coronavirus disease 2019 (COVID-19), which, in some individuals, progresses to acute respiratory distress syndrome characterized by aggressive inflammatory responses in the lower airways [reviewed in (*1*)]. Severe COVID-19 is due not only to direct effects of the virus but also, in part, to a misdirected host response with complex immune dysregulation of both innate and adaptive immune and inflammatory components (*2*, *3*). The COVID-19 pandemic has been met with an unprecedented research effort by the academia and the pharmaceutical industry. Nevertheless, by mid-2020, many aspects of COVID-19 immunopathogenesis still remain poorly characterized.

Most T cells respond in an adaptive fashion to peptide antigens governed by major histocompatibility complex (MHC) restriction, and the role of CD8 and CD4 T cell responses against COVID-19 has recently been demonstrated (*4*–*10*). However, the T cell compartment also encompasses several unconventional invariant T cell subsets that have innate-like functions (*11*). Mucosa-associated invariant T (MAIT) cells represent 1 to 10% of T cells in the circulation, have strong tissue homing characteristics, and are particularly abundant in the liver and lung [reviewed in (*12*)]. MAIT cells are activated by T cell receptor (TCR) recognition of microbial vitamin B_2_ (riboflavin) metabolites from a range of microbes presented by MHC-Ib–related protein 1 (MR1) molecules (*13*). However, some MAIT cell functions can be activated or coactivated by cytokines such as interleukin-18 (IL-18) and type I interferons (IFNs) (*14*, *15*). MAIT cells rapidly produce IFN-γ, tumor necrosis factor–α (TNF-α), and IL-17 and mediate effective cytolytic function dependent on granzyme B (GrzB) (*16*–*18*). This broad effector profile contributes to the role of MAIT cells in the protection against bacterial pulmonary infections (*19*–*21*), where MAIT cells have a role in recruiting adaptive T cells to the lung (*22*). MAIT cells in mucosal tissues have an IL-17/22–biased functional profile distinct from that of circulating cells (*23*) and can also be profibrogenic in chronic inflammatory disease (*24*).

Emerging evidence indicates that MAIT cells are innate-like sensors of viral infection; human MAIT cells are activated in response to several RNA viruses (*25*, *26*), expand during acute stages of HIV-1 infection (*27*), and may have a protective role in influenza virus infection as deduced from studies in murine models (*28*). On the basis of these distinct innate-like, tissue-homing, and proinflammatory characteristics of MAIT cells, we set out to study these cells in COVID-19. Our findings indicate that MAIT cells are engaged in the immune response against SARS-CoV-2 and suggest their possible involvement in COVID-19 immunopathogenesis.

## RESULTS

### Profound MAIT cell decline in the circulation and enrichment in the airways of COVID-19 patients

With the aim to study COVID-19 immunopathogenesis, 24 patients were prospectively recruited after admission to the Karolinska University Hospital as part of the Karolinska COVID-19 Immune Atlas project (*10*, *29*). Inclusion and exclusion criteria defined two groups of acute patients with moderate (AM) or severe (AS) disease matched with respect to other patient characteristics (table S1). Fourteen healthy donors (HDs) who were SARS-CoV-2 immunoglobulin G (IgG) seronegative and symptom-free at the time of sampling were included as controls. To minimize interexperimental variability and batch effects, all blood samples were acquired, processed, and analyzed fresh during three consecutive weeks in the spring of 2020 at the peak of the COVID-19 pandemic in Stockholm, Sweden.

**Fig. 1:**
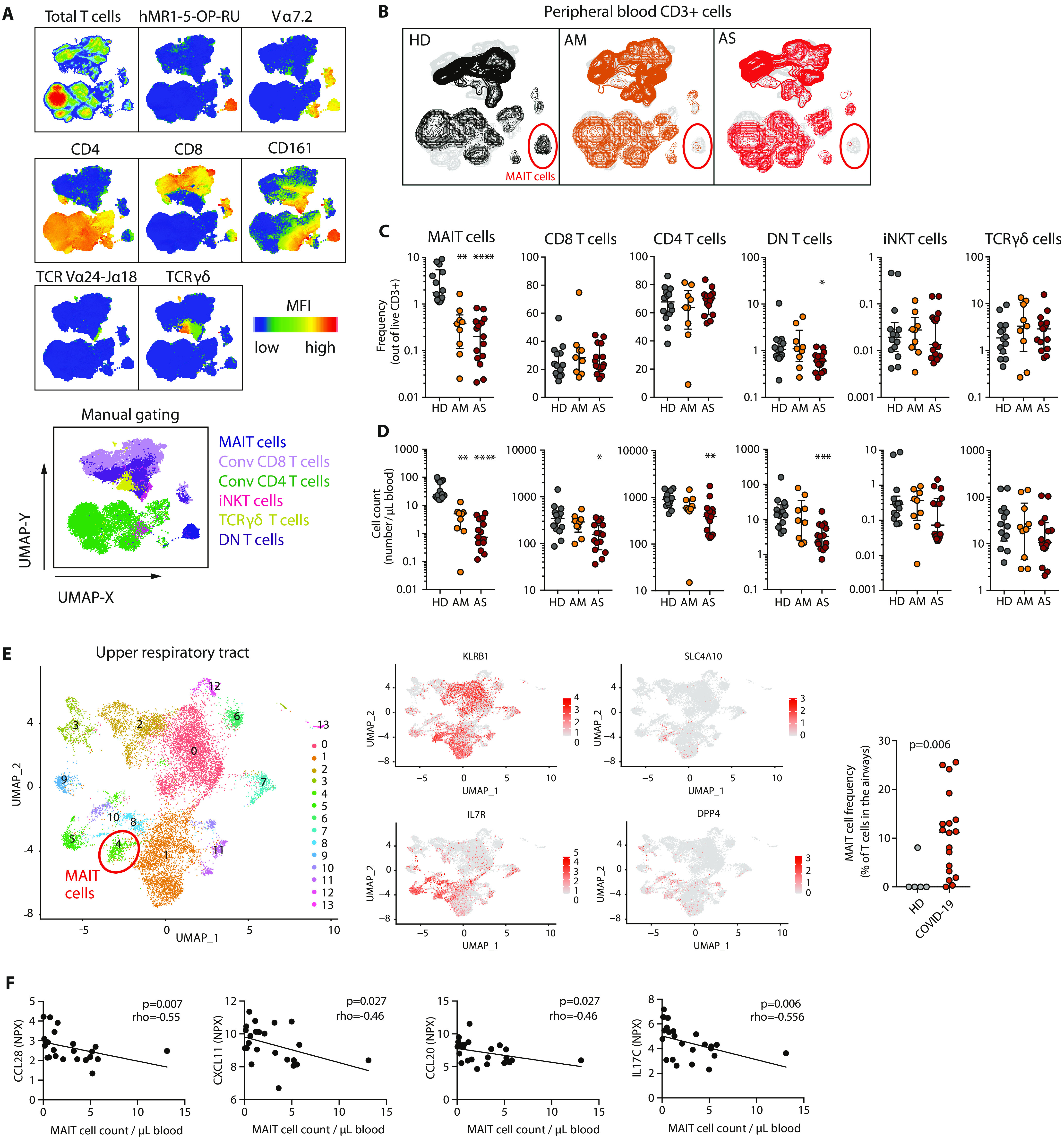
Profound and preferential decline of MAIT cells in blood of patients with COVID-19. (**A**) UMAP plots of total live CD3 T cells in peripheral blood showing expression of the indicated markers. Bottom: UMAP plots of total live CD3 T cells overlaid with the immune subsets identified by manual gating. (**B**) UMAP plots of total live CD3 T cells in peripheral blood colored according to the patient group: HD (*n* = 14), AM (*n* = 9), and AS (*n* = 15). CD3 T cells (20,000) per patient were down-sampled, barcoded according to the patient group, and concatenated. Red circle highlights the MAIT cell compartment. (**C**) Relative frequency [median ± interquartile range (IQR)] and (**D**) absolute counts (median ± IQR) of the indicated T cell subsets in peripheral blood. Each dot represents one donor. Nonparametric Kruskal-Wallis test and Dunn’s post hoc test were used to test for statistical differences between patient groups. *P < 0.05, **P < 0.01, ***P < 0.001, and ****P < 0.0001. (**E**) Left: UMAP plot displaying 13 clusters identified on the basis of gene expression levels of non–B lymphocytes in nasopharyngeal swabs (*n* = 4 HDs and *n* = 19 COVID-19 patients). Middle: UMAP plots of the transcripts KLRB1, SLC4A10, IL7R, and DPP4 used for MAIT cell identification. Right: Relative abundance of the MAIT cell–containing cluster (cluster 4) in nasopharyngeal swabs from HD and COVID-19 patients. Horizontal bars indicate medians. Each dot represents one donor. Nonparametric Mann-Whitney *U* test was used. (**F**) Spearman correlations between the absolute count of MAIT cells and cytokine and chemokine serum levels, expressed as NPX, in COVID-19 patients (*n* = 24). The Spearman correlation coefficient (rho) and the associated calculated *P* value (*P*) are indicated on each graph.

To study changes in conventional and unconventional T cell subsets in patients with AM and AS COVID-19 disease, a 22-parameter flow cytometry panel was designed to evaluate frequency, activation, homing, and functional phenotypes of MAIT cells, invariant natural killer T (iNKT) cells, CD4 and CD8 double-negative T (DNT) cells (*30*), γδ T cells, and conventional CD4 and CD8 T cells (fig. S1A). We initially analyzed the whole dataset through an unsupervised approach using Uniform Manifold Approximation and Projection (UMAP) analysis on CD3^+^ single live events in all patients and controls (*n* = 38). Projection of defining markers allowed visualization of the location of distinct T cell subsets on the UMAP topography (Fig. 1A), which was confirmed using manual gating. Projecting data from HD, AM, and AS subjects separately revealed a clear difference between patients and controls with severe reduction in the distinct topography defined by the MR1-5-OP-RU tetramer, suggesting loss of MAIT cells in COVID-19 (Fig. 1B). The profound decline in MAIT cell percentage (Fig. 1C) and absolute counts (Fig. 1D) in COVID-19 patients was confirmed by manual gating. The absolute count decline extended to conventional CD4 and CD8 T cell subsets and DNT cells, whereas iNKT cells and γδ T cells were largely unchanged. However, the MAIT cell lymphopenia was distinct in its severity and was pronounced already in the AM group where loss of overall T cell subsets was not significant (Fig. 1D and fig. S1B). The circulating MAIT cell pool comprises three subsets expressing CD8, CD4, or DN displaying some functional differences (*31*). The numerical decline of the DN MAIT cell subset was more marked than that of the CD8^+^ MAIT cell population (fig. S1C).

Analysis of a publicly available single-cell RNA sequencing (scRNAseq) dataset on nasopharyngeal samples (*32*) allowed identification of MAIT cells in the upper respiratory tract of COVID-19 patients and healthy control subjects using a combination of MAIT cell–defining transcripts (*KLRB1*, *SLC4A10*, *IL7R*, and *DPP4*) (Fig. 1E). The scRNAseq data indicated that MAIT cells were highly enriched within T cells infiltrating the airways of COVID-19 patients as compared with controls (Fig. 1E), consistent with the profound decline of the circulating MAIT cell pool in COVID-19 disease and a possible recruitment to this site. This pattern was corroborated by analysis of MAIT cells in a second published scRNAseq dataset on bronchoalveolar lavage fluid (*33*), which also confirmed the coexpression of *KLRB1*, *SLC4A10*, and *IL7R* in TRAV1-2^+^ cells (fig. S1D) (*34*). MAIT cell counts in blood correlated inversely with serum levels of CCL20 and CXCL11 in our cohort (Fig. 1F), the receptors for which (CCR6 and CXCR3, respectively) MAIT cells express at high levels (*35*). Furthermore, MAIT cell counts were inversely correlated with IL-17C levels in plasma, supporting a possible link between MAIT cell recruitment and lung epithelium inflammation (Fig. 1F) (*36*). These soluble factors were all increased in serum of COVID-19 patients compared with HD (fig. S1E). Together, these findings identify a pattern of profound and preferential decline of circulating MAIT cells in COVID-19 and suggest that this is at least partly caused by recruitment of MAIT cells to the airways.

### Distinct pattern of MAIT cell activation and loss of CXCR3 in COVID-19

**Fig. 2:**
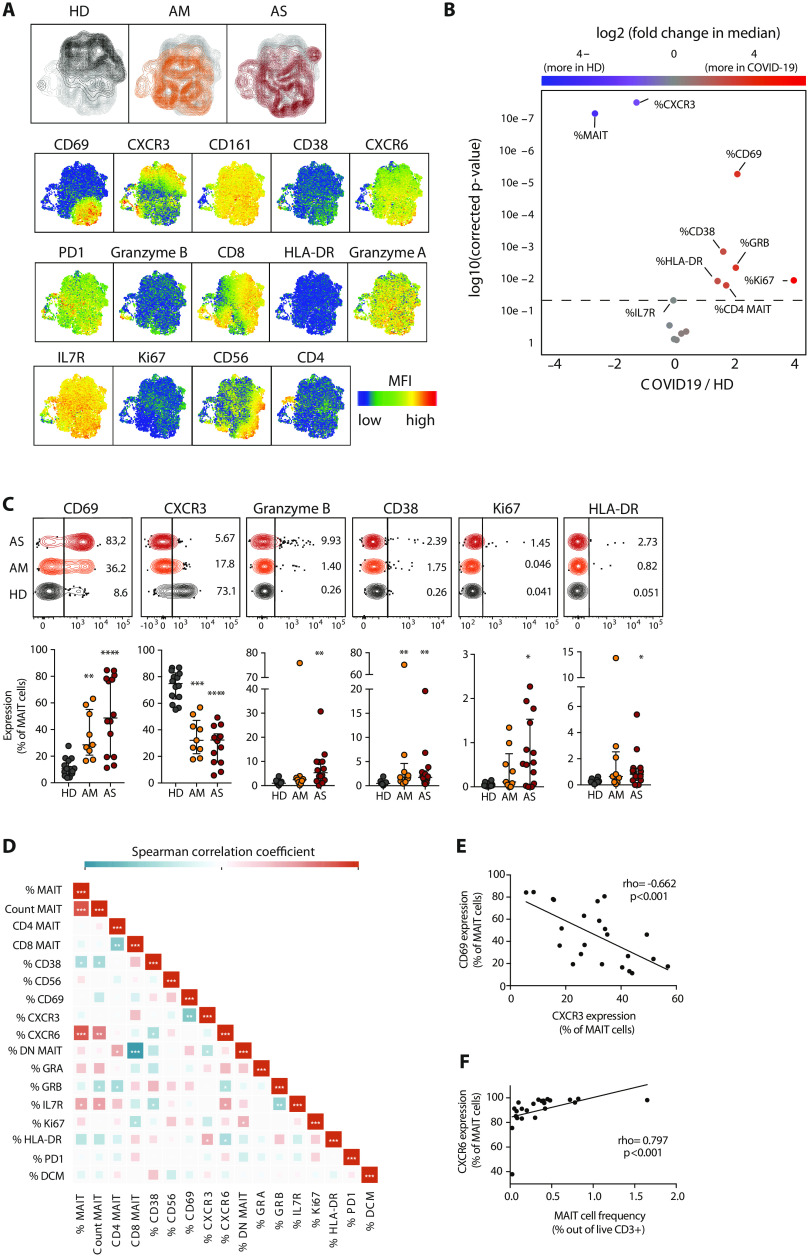
MAIT cell activation and decreased CXCR3 expression in peripheral blood of COVID-19 patients. (**A**) Top: UMAP plots of MAIT cells in peripheral blood showing the clustering of MAIT cells by patient group. A maximum of 200 MAIT cells per patient were down-sampled, labeled according to patient group, and concatenated. Bottom: UMAP plots of MAIT cells showing the expression of the indicated marker. (**B**) Volcano plot showing the log_2_ (fold change) in median expression of the indicated markers on MAIT cells between HDs (*n* = 14) and COVID-19 patients (*n* = 23). Significantly up-regulated or down-regulated markers (*P* < 0.05) are shown in red and blue, respectively. *P* values were calculated using Wilcoxon ranked exact test and adjusted to a false discovery rate of 5% using the Benjamini-Hochberg method. (**C**) Top: Illustrative concatenated flow cytometry plots showing the percentage of expression of the indicated phenotypic markers on MAIT cells by patient group. Bottom: Expression (median ± IQR) of the indicated markers on MAIT cells in HD (*n* = 14), AM (*n* = 9), and AS (*n* = 14) COVID-19. Nonparametric Kruskal-Wallis test and Dunn’s post hoc test were used to detect significant differences between groups. *P < 0.05, **P < 0.01, ***P < 0.001, and ****P < 0.0001. (**D**) Heatmap displaying pairwise Spearman correlations between MAIT cell phenotypic parameters in COVID-19 patients. Color indicates the strength of the correlation. *P < 0.05, **P < 0.01, and ***P < 0.001. (**E**) Spearman correlation between CXCR3 and CD69 expression on MAIT cells in COVID-19. (**F**) Spearman correlation between MAIT cell frequency and CXCR6 expression on MAIT cells in COVID-19. (E and F) The Spearman correlation coefficient (rho) and the associated calculated *P* value (*P*) are indicated on the graphs.

Next, we were interested to analyze MAIT cell characteristics in the context of COVID-19. Unsupervised analysis of MAIT cell flow cytometry phenotypes in all Atlas cohort patients and controls (*n* = 38) revealed a pattern of enhanced CD69 expression and diminished CXCR3 expression in both AM and AS COVID-19 patients (Fig. 2, A and B). The activated CD69^high^ phenotype was shared between AM and AS COVID-19 patients, although AS patients had slightly higher levels of GrzB and Ki67 than AM patients (Fig. 2C). Expression levels of PD-1, IL-7R, CXCR6, granzyme A (GrzA), and CD56 were similar between HD, AM, and AS groups (fig. S2A). Activation patterns were largely shared between CD8^+^ and DN MAIT cell subsets, although the CD8^+^ MAIT cell pool showed somewhat higher levels of activation (fig. S2B). The patterns with CD69 up-regulation and CXCR3 down-regulation were more pronounced in MAIT cells as compared with conventional CD4 and CD8 T cells (fig. S2C). Correlation analyses of the MAIT cell phenotype dataset (Fig. 2D) indicated that activation levels reflected by CD69 expression were inversely linked with CXCR3 expression (Fig. 2E), and MAIT cell percentages were directly correlated with CXCR6 expression (Fig. 2F).

Analysis of the scRNAseq dataset on nasopharyngeal samples (*32*) allowed characterization of the transcriptional profile of MAIT cells in the airways of COVID-19 patients (fig. S3). The CD69^+^CXCR3^−^ phenotype of MAIT cells in peripheral blood was reproduced at the transcriptional level in the airways. The transcriptional profile indicated that MAIT cells were the main subset of airway T cells expressing *IL17A*. This profile was paired with expression of *TNF* and an apparent lack of *IFNG* and *GZMB* transcripts. Together, these results indicate that the residual circulating MAIT cell pool is highly activated with lowered CXCR3 expression, a finding reproduced in the airways together with an *IL17A*^+^ transcriptional profile.

### MAIT cell characteristics associated with plasma viremia and disease outcome

**Fig. 3:**
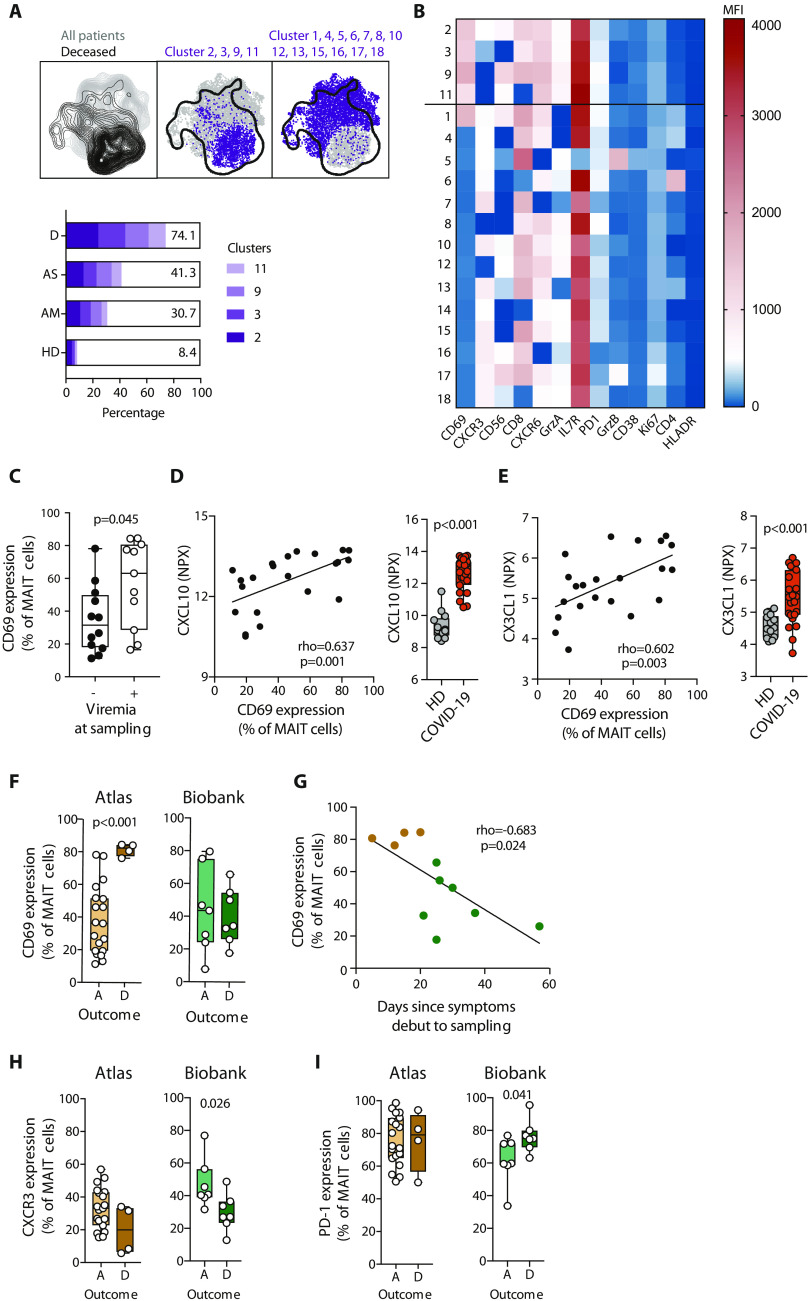
Associations between peripheral blood MAIT cell characteristics and COVID-19 outcome. (**A**) Top: UMAP plot of MAIT cells colored according to the outcome of the COVID-19 patients (left plot). Overlay of PhenoGraph clusters enriched in deceased (middle) or surviving (right) patients on the UMAP projection. Bottom: Proportion of MAIT cell clusters 2, 3, 9, and 11 for each patient group [HD, *n* = 14; AM, *n* = 9; AS, *n* = 14; deceased (D), *n* = 4]. (**B**) Heatmap of the median fluorescence intensity (MFI) of the phenotypic markers used to identify the 18 PhenoGraph MAIT cell clusters. The black line delimitates clusters enriched (top) or not (bottom) in deceased patients. (**C**) CD69 expression on MAIT cells in COVID-19 patients according to their viremia status at the time of sampling (−: negative, *n* = 12; +: positive, *n* = 11). (**D**) Left: Spearman correlation between CXCL10 serum level and CD69 expression on MAIT cells in COVID-19 patients (*n* = 23). Right: CXCL10 serum levels in HDs (*n* = 14) and COVID-19 patients (*n* = 24). (**E**) Left: Spearman correlation between CX3CL1 serum level and CD69 expression on MAIT cells in COVID-19 patients. Right: CX3CL1 serum levels in HD and COVID-19 patients (*n* = 23). (**F**) CD69 expression on MAIT cells in alive (A, *n* = 19) and deceased (D, *n* = 4) COVID-19 patients from the Atlas (left) and Biobank (right) cohorts. (**G**) Spearman correlation between CD69 expression on MAIT cells and the days since symptoms debut to sampling in deceased patients from the Atlas (brown) and the Biobank (green) cohorts. (**H**) CXCR3 and (**I**) PD-1 expression on MAIT cells in alive (A) and deceased (D) COVID-19 patients from the Atlas and Biobank cohorts. (D and E) Concentrations expressed as NPX (log_2_). (C to F, H, and I) Each dot represents one donor. Nonparametric Mann-Whitney test was used to detect significant differences between groups. (D, E, and G) Each dot represents one donor. The Spearman correlation coefficient (rho) and *P* value (*P*) are indicated on the graph.

We next investigated whether any characteristics of the MAIT cell compartment could be associated with the subsequent clinical outcome. Of the 24 patients sampled for the Karolinska COVID-19 Immune Atlas cohort and subjected to MAIT cell analyses, 20 recovered and were discharged, whereas 4 died in the hospital. Projection of the deceased patients on unsupervised UMAP analysis of MAIT cells from all patients and controls (*n* = 38) revealed a distinct distribution pattern (Fig. 3A, left plot). PhenoGraph clustering revealed four MAIT cell clusters overrepresented in the four patients who later died of COVID-19 (Fig. 3A). These PhenoGraph clusters were characterized by extraordinarily high CD69 expression and low or very low levels of CXCR3 (Fig. 3B and fig. S4). Within the entire Atlas patient group, CD69 was higher in patients with detectable plasma viremia (Fig. 3C and fig. S4) and positively correlated with serum levels of CXCL10 (Fig. 3D and fig. S4) and CX3CL1 (Fig. 3E and fig. S4).

We next analyzed the markers influencing PhenoGraph clustering in relation to outcome. The four patients who died at the hospital had significantly higher CD69 expression on their MAIT cells than patients who survived, suggesting an association between MAIT cell activation levels and clinical outcome (Fig. 3F, left). To explore this pattern further, we retrospectively identified another seven deceased intensive care unit (ICU) patients and seven matching ICU patients who were discharged alive (table S2), retrieved cryopreserved Biobank peripheral blood mononuclear cell (PBMC) samples from these patients, and stained their PBMCs using the same flow cytometry panel. In this retrospective sampling of severely ill patients, the association between CD69 levels and outcome was not reproduced (Fig. 3F, right). In comparing the first Atlas patient group with the second retrospective Biobank group, the Biobank patients were found to be sampled significantly later after symptom onset (25 versus 14 days, *P* < 0.001; table S3). Plotting data from all deceased patients in both cohorts revealed an inverse correlation between MAIT cell CD69 levels and days since symptom debut to sampling (Fig. 3G), raising the possibility that high levels of MAIT cell activation early in disease may be associated with immunopathogenesis and poor outcome.

In addition to high CD69, low CXCR3 and slightly higher PD-1 expression also characterized the PhenoGraph clusters associated with poor outcome (Fig. 3B). The four patients of the Atlas cohort who died at the hospital tended to have lower CXCR3 expression on their MAIT cells than patients who survived, and this pattern reached significance in the Biobank cohort (Fig. 3H). A similar pattern was observed with high PD-1 expression on MAIT cells, which was associated with poor outcome in the Biobank cohort (Fig. 3I). Together, these findings suggest that activation and chemokine receptor expression in MAIT cells are associated with disease severity and may be associated with clinical outcome of COVID-19 disease.

### Recovery of the MAIT cell compartment in COVID-19 convalescent individuals

**Fig. 4:**
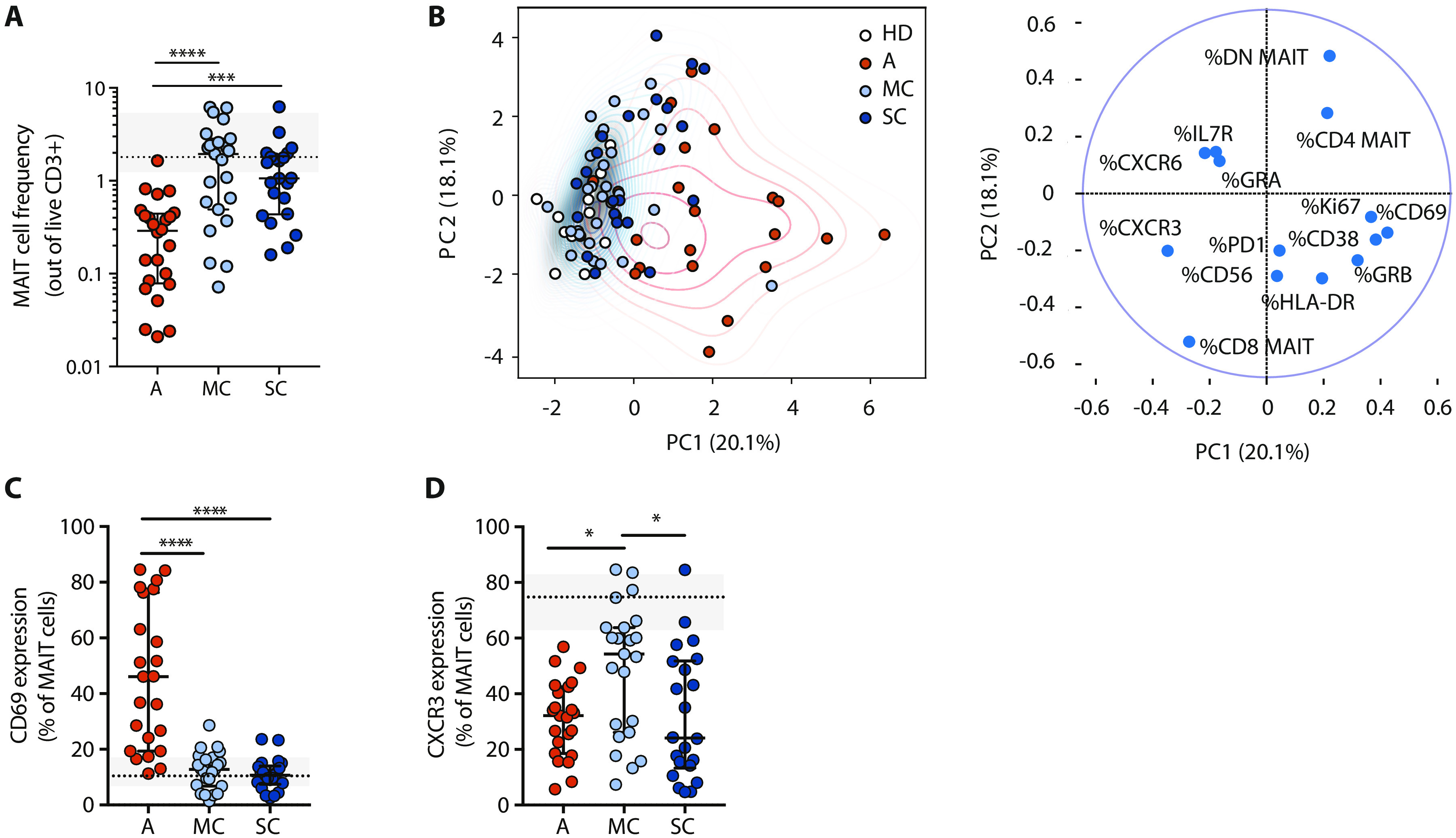
Recovery of peripheral blood MAIT cells in convalescent COVID-19. (**A**) MAIT cell peripheral blood frequency (median ± IQR) in all acute (A, *n* = 23), mild convalescent (MC, *n* = 23), and severe convalescent (SC, *n* = 22) COVID-19 patients. MAIT cell frequency (median ± IQR) in HDs (*n* = 14) is shown in gray. (**B**) Left: PCA showing the distribution and segregation of MAIT cell populations in HD (white dots), A (red dots, *n* = 23), MC (light blue, *n* = 23), and SC (dark blue, *n* = 22) COVID-19 patients. Right: PCA biplot representing the influence of each parameter on principal components 1 (PC1) and 2 (PC2). (**C**) Graph illustrating CD69 and (**D**) CXCR3 expression on MAIT cells in A, MC, and SC patient groups. Expression in HD (median ± IQR) is shown in gray. (A, C, and D) Each dot represents one patient. Nonparametric Kruskal-Wallis test and Dunn’s post hoc test were used to detect significant differences between the acute and convalescent groups. *P < 0.05, ***P < 0.001, and ****P < 0.0001.

Some chronic viral diseases are associated with partial loss of MAIT cells in the circulation, which may be persistent with failure to recover when viremia is suppressed or cleared by treatment (*37*–*39*). To determine the ability of MAIT cells to recover after COVID-19, we analyzed peripheral blood samples drawn from patients recovering from mild disease (MC) (*n* = 23) and from patients in the convalescent phase after severe COVID-19 (SC) (*n* = 22) within 1 to 6 weeks from resolution of disease (table S1). Compared with the pooled Atlas AM and AS patients groups (all acute, A), both MC and SC groups had MAIT cell frequencies, determined as percentage of T cells, similar to those of the HD group (Fig. 4A). The phenotypic flow cytometry characteristics of MAIT cells in the HD, A, MC, and SC groups were analyzed using principal components analysis (PCA) (Fig. 4B). The convalescent groups were largely overlapping with the HD group and distinct from the acute patients, where PC1 separated the acute group based on expression of activation markers. Some convalescent individuals appeared distinct from the HD group and were separated primarily by the relative representation of CD8^+^ and DN MAIT cell subsets contributing to PC2. Direct comparison of groups indicated that CD69 levels were largely normalized in convalescent patients (Fig. 4C). In contrast, CXCR3 levels were still suppressed, in particular in the SC group, raising the possibility that low CXCR3 expression may be a persistent alteration in MAIT cells after COVID-19 (Fig. 4D). CD38 and other activation markers tended to normalize (fig. S5). Together, these results indicate that MAIT cells recover in the circulation within weeks of resolution of COVID-19 symptoms, although some phenotypical perturbations still persist.

## DISCUSSION

MAIT cells play a significant role in the immune defense against microbial infections in mucosal barriers via TCR-mediated recognition of MR1-presented riboflavin metabolites. However, MAIT cells also function as innate sensors of inflammation and viral infection via activation by cytokines such as IL-18 and IFN-α (*12*). We here found that MAIT cells are highly activated in COVID-19 and decline sharply in numbers in the circulation already in moderate disease in a manner correlated with serum levels of several chemokines. The MAIT cell lymphopenia was more profound than that observed for conventional CD8 and CD4 T cells and other unconventional T cell subsets. Findings in two separate scRNAseq datasets indicated that MAIT cells are highly enriched among T cells infiltrating the airways during COVID-19.

MAIT cell activation levels (CD69^high^) were associated with detectable plasma viremia and correlated with increased serum levels of CXCL10 and CX3CL1. MAIT cell activation may be associated with down-regulation of CXCR3, as indicated by the inverse correlation between CD69 and CXCR3. The CXCR3^low^ phenotype seems more stable and was seen also in the mucosal scRNAseq data. Both these MAIT cell phenotypes (CD69^high^ and CXCR3^low^) were associated with poor clinical outcome among COVID-19 patients as assessed in the present cohort. One should bear in mind, however, that the number of patients studied here is small, and these MAIT cell phenotypes should not be interpreted as predictive biomarkers of poor outcome. Nevertheless, these associations and patterns support a model where MAIT cell activation may be part of the broader virus replication-driven type I IFN response (*40*).

The interpretation of preferential recruitment of MAIT cells to the inflamed airways gains further support from the distinct tissue homing profile of MAIT cells with expression of several relevant chemokine receptors, such as CCR6, CXCR6, CCR5, and CXCR3 (*35*). This notion has further support in the literature because activation and homing to sites of inflammation is consistent with observations in obesity and diabetes (*41*), multiple sclerosis (*42*), and in inflammatory bowel disease (*43*). Moreover, recent findings from bacterial pathogenesis indicate that the innate-like responsiveness of MAIT cells is important in initiation and amplification of the hyper-inflammatory response to bacterial superantigens (*44*, *45*). A notable pattern in the airway scRNAseq data was that the MAIT cell cluster displayed an IL-17A–biased profile and was the main T cell cluster expressing *IL17A* transcript. Previous studies have shown that mucosal tissue MAIT cells have an IL-17–biased proinflammatory profile with less IFN-γ expression (*23*, *46*) and can contribute to IL-17–mediated inflammation during bacterial pneumonia (*47*). The setting of COVID-19 is, however, quite distinct in that SARS-CoV-2 infection of the lung is unlikely to give rise to MR1-presented antigens. The MAIT cell response is thus more likely to be IFN- and cytokine-activated, although involvement of MR1-restricted autoantigens or secondary bacterial infection or microbiota-derived antigens cannot be excluded at this time.

During the time this report was in revision, reports have emerged in print or preprint with complementary findings addressing some aspects of the involvement of MAIT cells in COVID-19. Consistent with our findings, the reports by Kuri-Cervantes *et al.* (*9*), Jouan *et al.* (*48*), and Flament *et al.* (*49*) all observe a strong decline in MAIT cell percentages in the circulation. Together with our observation of strong and preferential absolute MAIT cell count decline, as well as the data pointing to enrichment of MAIT cells in the airways [this study and (*48*)], and very high level of MAIT cell activation [this study, (*48*), and (*49*)], all point to the strong response of this T cell subset in COVID-19. We and Flament *et al.* (*49*) see similar patterns where very strong MAIT cell activation is associated with severe outcome and death. Whereas this may seem to be in contrast to findings by Jouan *et al.* (*48*) suggesting possible benefit of MAIT cell activation, this discrepancy may potentially be explained by differences in study patient characteristics as indicated by relatively modest MAIT cell activation and few deaths in their study (*48*). Furthermore, activation as assessed by CD69 expression in peripheral blood MAIT cells may be higher early in disease and decline over time [this study and (*48*)]. Flament *et al.* (*49*) report activation of MAIT cells in vitro after exposure to SARS-CoV-2–infected macrophages. These data, together with our observation of higher activation of MAIT cells in patients with detectable plasma viremia, support a model where MAIT cells are activated in response to virus in an IFN- and IL-18–dependent manner. Together, in our view, these independent datasets collectively support a model where the strong activation and recruitment of MAIT cells to the airways is part of the response against SARS-CoV-2 infection. However, in individuals where this response is excessive, it may turn detrimental to the host and become a component of the pathological inflammatory process in COVID-19, where the IL-17A bias of mucosal MAIT cells may be important.

Persistent loss of MAIT cells can have detrimental long-term consequences for immune defense against microbial disease and immune homeostasis at barrier sites (*50*, *51*). Thus, it is interesting and promising that our findings indicate that MAIT cells seem to recover in convalescent COVID-19 patients, even within weeks from resolution of symptoms. This pattern is compatible with recruitment to the airways rather than physical loss of MAIT cells during active disease. It is possible that, as inflammation is resolved, the MAIT cells are again released to the circulation. With regard to the phenotypical MAIT cell characteristics, most seem to normalize in convalescence. However, CXCR3 expression remains suppressed, and some convalescent donors have a disturbed balance of CD8^+^ and DN MAIT cell subsets. The CXCR3^low^ phenotype is more stable than the more transient CD69^high^ phenotype. Overall, the relatively rapid recovery of the MAIT cell compartment bodes well for the ability of these individuals to control future microbial infections, although this topic will require dedicated longitudinal studies.

Loss of detectable CXCR3 expression in MAIT cells seems to be linked to activation as indicated by the inverse correlation with CD69 expression. However, a range of other chemokine receptors might be involved in recruitment of MAIT cells to inflamed airways. This includes CXCR6, which is consistently expressed by MAIT cells in HDs and largely remains expressed in COVID-19 albeit with somewhat lower expression in patients with reduced MAIT cell frequencies (Fig. 2F). Note that the gene encoding CXCR6 is one of six genes located within the 3p21.31 locus strongly associated with severe COVID-19 in a recent genome-wide association study (*52*). The role of CXCR6, as well as of CCR5 and CXCR4, in MAIT cell recruitment in COVID-19, and in COVID-19 in general, is currently unclear and should be topics of future investigation.

The current study presents evidence for a multifaceted role of MAIT cells in COVID-19 disease. This role is distinct from that of adaptive conventional T cells because MAIT cells do not recognize human leukocyte antigen (HLA)–presented peptide antigens. Their antiviral function is therefore probably innate-like and intrinsically less effective than the MHC-restricted CD8 T cell response on a per cell basis. On the other hand, MAIT cells are available to respond rapidly in large numbers without clonal expansion, and one may speculate that their antiviral effect could be important in early infection. Furthermore, MAIT cells have been shown in murine models to have a role in recruiting adaptive T cells to the lung (*22*). The findings of the current study open the possibility that the rapid and proinflammatory MAIT cell response may also play a role in the pathological inflammation of COVID-19.

Last, it is important to note that our study has several limitations. COVID-19 is heterogeneous in its presentation with a wide range of disease severity, symptoms, and kinetics of disease. Although the patient groups studied here were all well defined, they still do not reflect the full complexity of the disease. Furthermore, the cross-sectional design does not capture the full disease dynamics. In particular, sampling very early after infection or symptom debut would be valuable in future studies. Nevertheless, despite these limitations, the current study supports a role for MAIT cells in COVID-19 and opens new avenues to be explored for better understanding of the immunopathogenesis of this disease.

## MATERIALS AND METHODS

### Patient characteristics

SARS-CoV-2–infected patients 18 to 78 years old (*n* = 69) with acute COVID-19 disease admitted to the Karolinska University Hospital, Stockholm, Sweden or followed up in convalescent phase were recruited to the study. Healthy controls (*n* = 14) were SARS-CoV-2 IgG seronegative at the time of inclusion, matched for sex, and in the same age range as the COVID-19 patients. Patients in the Karolinska COVID-19 Immune Atlas cohort were sampled 5 to 24 days after symptom debut and 0 to 8 days after hospital admission in the acute phase. Those classified as having AM COVID-19 disease (*n* = 9) had oxygen saturation of 90 to 94% or were receiving oxygen (0.5 to 3 liters/min) at the time of inclusion. Patients with AS COVID-19 (*n* = 15) required either >10 liters/min of oxygen at sampling or invasive mechanical ventilation and were treated in one of the Karolinska University Hospital ICUs or a high-dependency unit. For both AM and AS groups, patients with current malignant disease or ongoing immunomodulatory treatment before hospitalization were excluded. Samples from individuals in the convalescent phase after severe disease (SC) (*n* = 22) were collected 42 to 58 days after disease onset, corresponding to 3 to 21 days after resolution of symptoms (100% were antibody-seropositive for SARS-CoV-2). Samples obtained from individuals in the convalescent phase after mild disease (*n* = 23) were collected 49 to 64 days after disease onset, corresponding to 25 to 53 days after resolution of symptoms. Samples from the COVID-19 Biobank cohort were PBMC from ICU patients cryopreserved in liquid nitrogen and thawed immediately before staining (*n* = 14). The severity of the disease was also graded with the National Institutes of Health (NIH) Ordinal Scale (*53*) and sequential organ failure assessment (SOFA) score at the sampling (*54*). For detailed clinical information, see tables S1 and S2. The study was approved by the Swedish Ethical Review Authority, and all patients gave informed consent.

### Flow cytometry and antibodies

The following antibodies were used for staining: CD69-BUV395 (clone FN50), CD38-BUV496 (clone HIT2), CD56-BUV737 (clone NCAM16.2), CD3-BUV805 (clone UCHT1), CD14-V500 (clone M5E2), CD19-V500 (clone HIB19), Vα24-BV750 (clone L243), CD161-PE-Cy5 (clone DX12), and GrzB-AF700 (clone GB119) from BD Biosciences; PD1-BV421 (clone EH12.2H7), CD8-BV570 (clone RPA-T8), IL7R-BV605 (clone A019D5), CXCR3-BV650 (clone G025H7), CD4-BV711 (clone OKT4), HLA-DR-BV785 (clone L243), Ki-67-AF488, GrzA-PercCP-Cy5.5 (clone CB9), TCRγδ-PE/Dazzle564 (clone B1), Vα7.2-PE-Cy7 (clone 3C10), and CXCR6-AF647 (clone K041E5) from BioLegend. Phycoerythrin (PE)–conjugated 5-OP-RU–loaded human MR1 tetramer (NIH Tetramer Core Facility) was used for the identification of MAIT cells. LIVE/DEAD Fixable Near-Infrared Dead Cell Stain Kit (Thermo Fisher Scientific) was used for the staining of dead cells. Cells were first stained with the hMR1-5-OP-RU tetramer for 30 min at room temperature (RT) before extracellular staining for another 20 min at 4°C in phosphate-buffered saline, 2 mM EDTA, and 2% fetal bovine serum. After washing, cells were fixed and permeabilized in the BD Cytofix/Cytoperm Fixation/Permeabilization Kit (BD Biosciences) for 30 min at 4°C and stained intracellularly in 1× BD Perm/Wash buffer (BD Biosciences) for 30 min at 4°C. After washing, cells were fixed for 2 hours at RT in 1% paraformaldehyde (PFA) before acquisition. Samples were acquired on a BD FACSymphony A5 flow cytometer (BD Biosciences) and analyzed with FlowJo software version 10.6.2 (FlowJo LLC). Single-stained compensation beads (BD Biosciences) were used to calculate compensation matrix before sample acquisition. Stainings were performed on freshly isolated (Atlas and convalescent cohorts) or cryopreserved (Biobank cohort) PBMCs.

### Serum proteomics

Sera were evaluated for soluble factors using proximity extension assay technology (Olink AB, Uppsala). All sera were heat-inactivated (56°C for 30 min) before analysis. Results are presented as Normalized Protein eXpression (NPX) on a log_2_ scale that allows relative quantification of each individual marker across samples.

### Trucount

Absolute counts in whole blood were assessed by flow cytometry using BD Multitest six-color T-, B-, and NK-cell (TBNK) reagents in association with BD Trucount tubes (BD Biosciences) following the manufacturer’s instructions. Samples were fixed 2 hours at RT in 1% PFA before acquisition on the BD FACSymphony (BD Biosciences). CD3^+^ T cells were gated out of CD45^+^CD14^−^CD15^−^CD19^−^ cells, and the number of events obtained was used to determine the absolute CD3 counts as follows: (number of CD3^+^ events acquired × number of beads per tubes)/(number of beads events acquired × sample volume in microliters). The absolute count of each T cell subset analyzed was subsequently calculated using their frequencies out of total CD3^+^ cells. When changes in absolute count were expressed as percentages, they were calculated as follows: (median of absolute count in HD − median of absolute count in patients)/(median of absolute count in HD)*100.

### scRNAseq analysis

Publicly available scRNAseq datasets were analyzed using standard Seurat (3.2.0) workflow. Briefly, data files from Chua *et al.* (*32*) (https://doi.org/10.6084/m9.figshare.12436517) and Liao *et al.* (*33*) (https://covid19-balf.cells.ucsc.edu) datasets were downloaded. Cells annotated as non–B lymphocytes were selected, clustered, and projected on UMAP. The clusters containing MAIT cells were identified on the basis of expression of *KLRB1*, *DPP4*, *IL7R*, and *SLC4A10*, as well as *TRAV1-2* transcripts when available in the data. MAIT cell frequency among T cells was then estimated as the percentage of cells belonging to the MAIT-containing cluster among all cells annotated as T cells.

### UMAP and PhenoGraph analysis

FCS3.0 files exported from BD FACSDiva software were imported into FlowJo software, and automated compensation matrix was generated using the acquired single-stained compensation beads. To generate the UMAP, samples were first down-sampled using the FlowJo plugin Downsample (V2.0.0), barcoded with patient group and patient outcome, and concatenated. The FlowJo plugin UMAP (V2.2) was run on the resulting flow cytometry standard (FCS) file using the default settings (distance function: Euclidean, nearest neighbors: 15, and minimum distance: 0.5) and including all the compensated parameters and forward scatter (FSC) and side scatter (SSC) measurements. For cluster identification, the FlowJo plugin PhenoGraph (V2.4) was run on the resulting UMAP using the default settings (nearest neighbors *K* = 30) and including the following parameters: CD69, CD38, HLA-DR, PD1, CXCR3, CXCR6, IL7R, Ki-67, CD56, CD4, CD8, GrzB, and GrzA.

### Principal components analysis

PCA was performed in Python, using scikit-learn 0.22.1. Phenotypic data obtained from flow cytometry for each cell subset were normalized using sklearn.preprocessing.StandardScaler, and PCA was computed on the resulting *z* scores.

### Statistical analysis

Prism V7.0 (GraphPad Software) and python were used for statistical analysis. Statistically significant differences between unpaired groups were determined using nonparametric Mann-Whitney test for two-group comparison or Kruskal-Wallis test followed by Dunn’s post hoc correction when more than two groups were compared. Two parameter correlations were evaluated using the Spearman correlation. Correlation heatmaps were generated in Python using the pingouin package v0.3.6 (https://pingouin-stats.org) for computing Spearman and rank-biserial correlations as well as the associated *P* values.

## SUPPLEMENTARY MATERIALS

immunology.sciencemag.org/cgi/content/full/5/51/eabe1670/DC1 Fig. S1. Gating strategy, MAIT cell abundance, and alteration of soluble factors in COVID-19 patients. Fig. S2. Phenotypic alterations of peripheral blood MAIT cells and other T cell subsets in COVID-19 patients. Fig. S3. Transcriptional profiling of the MAIT cell compartment in the upper respiratory tract. Fig. S4. Associations and correlations of MAIT cell count and phenotype with clinical parameters and cytokine and chemokine levels in serum. Fig. S5. Phenotypic comparison of peripheral blood MAIT cells in acute and convalescent COVID-19 patients. Table S1. Demographic and clinical characteristics of the acute COVID-19 Atlas cohort, HDs, and convalescent patients. Table S2. Demographic and clinical characteristics of the COVID-19 Biobank cohort patients. Table S3. Demographic and clinical data comparison between the acute COVID-19 Atlas cohort and the Biobank cohort. Table S4. Raw data file (Excel spreadsheet).

## Appendix

View/request a protocol for this paper from *Bio-protocol*.
